# ^61^Cu-PSMA–Targeted PET for Prostate Cancer: From Radiotracer Development to First-in-Human Imaging

**DOI:** 10.2967/jnumed.123.267126

**Published:** 2024-09

**Authors:** Tais Basaco Bernabeu, Rosalba Mansi, Luigi Del Pozzo, Sandra Zanger, Raghuvir H. Gaonkar, Lisa McDougall, Francesco De Rose, Leila Jaafar-Thiel, Michael Herz, Matthias Eiber, Gary A. Ulaner, Wolfgang A. Weber, Melpomeni Fani

**Affiliations:** 1 Division of Radiopharmaceutical Chemistry, University Hospital Basel, Basel, Switzerland;; 2 Nuclidium AG, Basel, Switzerland;; 3 Department of Nuclear Medicine, Technical University of Munich, Munich, Germany;; 4 Bavarian Cancer Research Center, Munich, Germany;; 5 Molecular Imaging and Therapy, Hoag Family Cancer Institute, Irvine, California; and; 6 Departments of Radiology and Translational Genomics, University of Southern California, Los Angeles, California

**Keywords:** PSMA, copper-61, prostate cancer, PET, theranostics

## Abstract

The demand for PET tracers that target prostate-specific membrane antigen (PSMA) continues to increase. Meeting this demand with approved ^68^Ga- and ^18^F-labeled PSMA tracers is challenging outside of major urban centers. This is because the short physical half-life of these radionuclides makes it necessary to produce them near their sites of usage. To overcome this challenge, we propose cyclotron-produced ^61^Cu for labeling PSMA PET tracers. ^61^Cu can be produced on a large scale, and its 3.33-h half-life allows shipping over considerably longer distances than possible for ^68^Ga and ^18^F. Production of true theranostic twins using ^61^Cu and the β^−^-emitter ^67^Cu is also feasible. **Methods:** PSMA-I&T (DOTAGA-(l-y)fk(sub-KuE)) and its derivative in which the DOTAGA chelator was replaced by NODAGA (NODAGA-(l-y)fk(sub-KuE)), herein reported as DOTAGA-PSMA-I&T and NODAGA-PSMA-I&T, respectively, were labeled with ^61^Cu and compared with [^68^Ga]Ga-DOTAGA-PSMA-I&T, [^68^Ga]Ga-NODAGA-PSMA-I&T, [^68^Ga]Ga-PSMA-11, and [^18^F]PSMA-1007. In vitro (lipophilicity, affinity, cellular uptake, and distribution) and in vivo (PET/CT, biodistribution, and stability) studies were performed in LNCaP cells and xenografts. Human dosimetry estimates were calculated for [^61^Cu]Cu-NODAGA-PSMA-I&T. First-in-human imaging with [^61^Cu]Cu-NODAGA-PSMA-I&T was performed in a patient with metastatic prostate cancer. **Results:** [^61^Cu]Cu-DOTAGA-PSMA-I&T and [^61^Cu]Cu-NODAGA-PSMA-I&T were synthesized with radiochemical purity of more than 97%, at an apparent molar activity of 24 MBq/nmol, without purification after labeling. In vitro, natural Cu (^nat^Cu)-DOTAGA-PSMA-I&T and ^nat^Cu-NODAGA-PSMA-I&T showed high affinity for PSMA (inhibitory concentration of 50%, 11.2 ± 2.3 and 9.3 ± 1.8 nM, respectively), although lower than the reference ^nat^Ga-PSMA-11 (inhibitory concentration of 50%, 2.4 ± 0.4 nM). Their cellular uptake and distribution were comparable to those of [^68^Ga]Ga-PSMA-11. In vivo, [^61^Cu]Cu-NODAGA-PSMA-I&T showed significantly lower uptake in nontargeted tissues than [^61^Cu]Cu-DOTAGA-PSMA-I&T and higher tumor uptake (14.0 ± 5.0 percentage injected activity per gram of tissue [%IA/g]) than [^61^Cu]Cu-DOTAGA-PSMA-I&T (6.06 ± 0.25 %IA/g, *P* = 0.0059), [^68^Ga]Ga-PSMA-11 (10.2 ± 1.5 %IA/g, *P* = 0.0972), and [^18^F]PSMA-1007 (9.70 ± 2.57 %IA/g, *P* = 0.080) at 1 h after injection. Tumor uptake was also higher for [^61^Cu]Cu-NODAGA-PSMA-I&T at 4 h after injection (10.7 ± 3.3 %IA/g) than for [^61^Cu]Cu-DOTAGA-PSMA-I&T (4.88 ± 0.63 %IA/g, *P* = 0.0014) and [^18^F]PSMA-1007 (6.28 ± 2.19 %IA/g, *P* = 0.0145). Tumor-to-nontumor ratios of [^61^Cu]Cu-NODAGA-PSMA-I&T were superior to those of [^61^Cu]Cu-DOTAGA-PSMA-I&T and comparable to those of [^68^Ga]Ga-PSMA-11 and [^18^F]PSMA-1007 at 1 h after injection and increased significantly between 1 and 4 h after injection in most cases. Human dosimetry estimates for [^61^Cu]Cu-NODAGA-PSMA-I&T were similar to the ones reported for ^18^F-PSMA ligands. First-in-human imaging demonstrated multifocal osseous and hepatic metastases. **Conclusion:** [^61^Cu]Cu-NODAGA-PSMA-I&T is a promising PSMA radiotracer that compares favorably with [^68^Ga]Ga-PSMA-11 and [^18^F]PSMA-1007, while allowing delayed imaging.

## Visual Abstract

**Figure:**
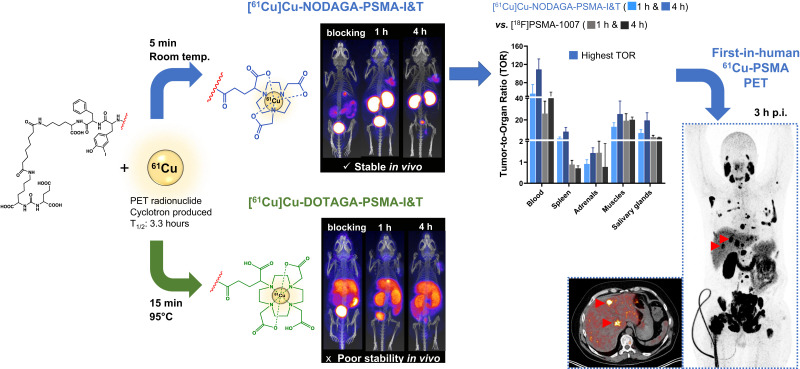

PET that targets prostate-specific membrane antigen (PSMA) continues to grow in usage. It has shown clinical value in the initial staging of newly diagnosed high-risk prostate cancer, localization of disease sites in patients with biochemical recurrence, and identification of appropriate patients for PSMA-targeted radiopharmaceutical therapy (1–4). Several PSMA-targeted PET tracers have been developed, with most bearing Glu-urea-Lys as the binding motif (5*,*^6^). Among them, [^68^Ga]Ga-PSMA-11 (^68^Ga-gozetotide), [^18^F]DCFPyL (^18^F-piflufolastat), and [^18^F]rhPSMA-7.3 (^18^F-flotufolastat) are approved by the Food and Drug Administration. With PSMA-targeted radiopharmaceutical therapy becoming an important option (7*,*^8^), the demand for PSMA PET scans is expected to expand rapidly across the globe (9).

So far, only ^68^Ga- and ^18^F-labeled PSMA tracers have been used (5*,*^6^). However, the relatively short half-life (t_1/2_) of ^68^Ga (t_1/2_, 68 min) and of ^18^F (t_1/2_, 110 min) limits the typical geographic distribution range of these radiotracers to about 160 km (100 miles). Because not all medical centers have radiochemistry facilities or exist close to radiotracer production sites, there are substantial gaps in geographic coverage for these PET tracers.

We propose, as an alternative, cyclotron-produced ^61^Cu (t_1/2_, 3.33 h; 61% β^+^-fraction; mean positron energy, 500 keV; maximum positron energy, 1,216 keV) for labeling PSMA PET tracers. ^61^Cu has the following advantages as a radioisotope for PET imaging. First, ^61^Cu can be produced in cyclotrons on a large scale, similar to ^18^F, and combines the attractive logistics of centralized radiotracer production with chelator-based radiochemistry, similar to ^68^Ga (10). Transport by land to remote PET facilities is possible up to a radius of 300 km (186 miles) within 1 t_1/2_. Fewer than 10 production sites would be required to supply ^61^Cu or ^61^Cu-labeled tracers to most populous regions in the continental United States or Europe (11*,*^12^). Second, the longer physical t_1/2_ of ^61^Cu enables delayed imaging when image contrast will be higher, because the radioactivity from PSMA ligands is cleared only slowly from the tumor cells. Third, ^61^Cu can be paired with β^−^-emitting ^67^Cu to create true theranostic twins for imaging and therapy agents.

Despite the advantages of ^61^Cu, it has not been widely used for the development of PET tracers, mainly because of its lack of availability, low radionuclide purity, and low yields (10). These issues have been addressed recently (13*,*^14^). In 2020, Svedjehed et al. (14) developed an automated procedure for isolating [^61^Cu]CuCl_2_ from cyclotron-irradiated Ni targets. The increased availability of ^61^Cu opens opportunities for its use.

We report herein the development of the first ^61^Cu-labeled PSMA-targeted tracers. We used PSMA-I&T (DOTAGA-(I-y)fk(sub-KuE)), which was evaluated in a phase 2 clinical trial labeled with ^64^Cu (NCT05653856). In parallel, we developed the new NODAGA derivative of PSMA-I&T (NODAGA-(l-y)fk(sub-KuE)), based on our previous work demonstrating advantages of the chelator NODAGA over DOTA- or cyclam-based chelators for ^64^Cu (15*,*^16^). The 2 derivatives, herein reported as DOTAGA-PSMA-I&T and NODAGA-PSMA-I&T, were used for ^61^Cu radiotracer development, in vitro and in vivo characterization, and first-in-human imaging.

## MATERIALS AND METHODS

All information on the reagents, analytic methods, cell line, and experimental procedures are provided in the supplemental materials (supplemental materials are available at http://jnm.snmjournals.org).

### Production and Purification of [^61^Cu]CuCl_2_

[^61^Cu]CuCl_2_ was produced by irradiating natural nickel (^nat^Ni) electroplated on silver coins at 40 µA over 120 min in a GE Healthcare medical cyclotron at the University Hospital Zurich, Switzerland, followed by purification based on Svedjehed et al. (14). The process yielded approximately 1 GBq/mL [^61^Cu]CuCl_2_ in 0.05 M HCl. Details on the production, purification, and extraction of ^61^Cu will be published elsewhere.

### PSMA Radiotracers

The synthesis of [^61^Cu]Cu-DOTAGA-PSMA-I&T and [^61^Cu]Cu-NODAGA-PSMA-I&T and the quality control are described in the supplemental materials, together with the reference tracers [^68^Ga]Ga-DOTAGA-PSMA-I&T, [^68^Ga]Ga-NODAGA-PSMA-I&T, [^68^Ga]Ga-PSMA-11, and [^18^F]PSMA-1007 used for comparison.

### In Vitro Characterization: log*D*, Affinity, Cellular Uptake, and Distribution

The lipophilicity of [^61^Cu]Cu-DOTAGA-PSMA-I&T and [^61^Cu]Cu-NODAGA-PSMA-I&T was assessed by determining the distribution coefficient in a 1:1 mixture of 1-octanol–to–phosphate-buffered saline at pH 7.4, in comparison with all reference radiotracers. Inhibitory concentration of 50% (IC_50_) was assessed by competition binding on lymph node carcinoma of the prostate (LNCaP) using ((*S*)-1-carboxy-5-(4-(-^125^I-iodo-benzamido)pentyl)carbamoyl)-l-glutamic acid as the reference radioligand. Cellular uptake and distribution were assessed in LNCaP cells at 5, 15, 30, 60, and 120 min after exposure to the radioligand at 37°C, either alone or in the presence of 10 µM 2-(phosphonomethyl)pentanedioic acid to distinguish between specific and nonspecific uptake.

### Animal Studies

All animal experiments were conducted in accordance with Swiss animal welfare laws and regulations under license number 30515 granted by the Veterinary Office, Department of Health, Canton Basel-Stadt, Switzerland. Male athymic nude-*Foxn1^nu^/Foxn1^+^* mice (Envigo), 4–6 wk old, were inoculated subcutaneously on the shoulder with 10^7^ LNCaP cells, freshly suspended in a 1:1 ratio of sterile minimum essential medium with basal medium Eagle and Matrigel. The tumors were allowed to grow to a volume of approximately 200 mm^3^.

### Preclinical PET/CT Imaging

Dynamic PET scans 0–1 h after injection were acquired using the β-CUBE PET scanner (Molecubes) after intravenous administration of ^61^Cu-labeled tracers (100 µL/400 pmol/7–8 MBq), ^68^Ga-labeled tracers (100 µL/400 pmol/6–9 MBq), and [^18^F]PSMA-1007 (100 µL/70 pmol/15 MBq). In addition, static scans at 4 h after injection were acquired for the ^61^Cu-labeled tracers and [^18^F]PSMA-1007. CT scans were acquired in nano-SPECT/CT (Bioscan; Mediso). Mice were anesthetized with 1.5% isoflurane, and dynamic PET scans were acquired within 1 h after injection. The mice were euthanized by CO_2_ at 4 h after injection, the bladder was mechanically emptied, and static PET scans were acquired for 30 min. Details on image acquisition and reconstruction parameters are described in the supplemental materials.

### Biodistribution Studies

The biodistribution of [^61^Cu]Cu-DOTAGA-PSMA-I&T and [^61^Cu]Cu-NODAGA-PSMA-I&T (100 µL/200 pmol/2–3 MBq) was compared with that of [^68^Ga]Ga-DOTAGA-PSMA-I&T, [^68^Ga]Ga-NODAGA-PSMA-I&T, and [^68^Ga]Ga-PSMA-11 (100 µL/200 pmol/3–5 MBq), as well as [^18^F]PSMA-1007 (100 µL/70 pmol/15 MBq). This comparison was conducted at 1 h after injection for all radiotracers and at 4 h after injection for the ^61^Cu-labeled tracers and [^18^F]PSMA-1007. The specificity of the ^61^Cu-labeled tracers was assessed at 1 h after injection by blocking studies with 2-(phosphonomethyl)pentanedioic acid (100 µL/1.3 µmol) being injected 3–5 min before the injection of the radiotracer.

### In Vivo Metabolic Stability

The stability of [^61^Cu]Cu-DOTAGA-PSMA-I&T and [^61^Cu]Cu-NODAGA-PSMA-I&T was assessed by radio–reversed-phase high-performance liquid chromatography in urine and in liver and kidney homogenates from healthy BALB/c mice after injection of 100 µL/400 pmol/8–9 MBq of each radiotracer. Details are provided in the supplemental materials.

### Dosimetry

Additional biodistribution data were generated in healthy BALB/c mice using [^64^Cu]Cu-NODAGA-PSMA-I&T at 1, 4, 12, and 24 h after injection and combined with the data of [^61^Cu]Cu-NODAGA-PSMA-I&T at 1 and 4 h after injection. Non–decay-corrected biodistribution data for ^61^Cu (t_1/2_, 3.33 h) were used to generate time–activity curves for [^61^Cu]Cu-NODAGA-PSMA-I&T. OLINDA/EXM version 1.0 (Vanderbilt University) was used for the dosimetry estimates, as described in the supplemental materials.

### First-in-Human PET/CT Imaging

[^61^Cu]Cu-NODAGA-PSMA-I&T was produced at the Nuclear Medicine Department of the Klinikum Rechts der Isar (Technical University of Munich) for human use. It was applied according to §13.2b of the German pharmaceutical law, and the requirement to obtain consent for the retrospective data analysis was waived. The manufacture of the tracer was performed via an automated process through a GE Healthcare FASTlab 2 module. Details on the production and quality controls are described in the supplemental materials. A dose of 105 MBq/32 µg was administered intravenously to a patient with known metastatic prostate cancer before ^177^Lu-labeled PSMA radiopharmaceutical therapy. The patient was coinjected with 10 mg of furosemide (Lasix; Sanofi-Aventis). Imaging was performed 3 h after tracer administration on a Biograph Vision PET/CT scanner (Siemens Healthineers). Images were obtained from the skull to mid-thigh and reconstructed into multiplanar PET, CT, and fused PET/CT images. CT was used for attenuation correction.

### Statistics

Statistical analysis was performed by unpaired *t* testing with Welch correction using GraphPad Prism version 9 (GraphPad Software). *P* values of less than 0.05 were considered significant. All data were evaluated as mean ± SD.

## RESULTS

### ^61^Cu-PSMA Tracers: Radiochemistry and In Vitro Characterization

^61^Cu production by solid target irradiation of ^nat^Ni, followed by purification as described earlier, resulted in a 1.4- to 2.1-GBq yield and more than 99.99% radionuclidic purity at 12 h after purification. Details on production and quality control results of the [^61^Cu]CuCl_2_ solution used for radiolabeling will be published elsewhere. The analytic data and the in vitro properties of [^61^Cu]Cu-DOTAGA-PSMA-I&T and [^61^Cu]Cu-NODAGA-PSMA-I&T are summarized in Table 1.

**TABLE 1.** Analytic Data and In Vitro Properties of [^61^Cu]Cu-DOTAGA-PSMA-I&T and [^61^Cu]Cu-NODAGA-PSMA-I&T

Radioligand	RCP	t_R_ (min)	log*D*_pH7.4_	IC_50_ (nM)*	Internalized fraction (%)	Surface-bound fraction (%)
[^61^Cu]Cu-DOTAGA-PSMA-I&T	97.4 ± 2.3	7.2 ± 0.2	−2.69 ± 0.44	11.2 ± 2.3	13.3 ± 0.5	13.4 ± 0.8
[^61^Cu]Cu-NODAGA-PSMA-I&T	98.2 ± 1.9	7.0 ± 0.3	−2.95 ± 0.08	9.3 ± 1.8	11.7 ± 1.6	10.8 ± 1.8

* Determined using ^nat^Cu-DOTAGA-PSMA-I&T and ^nat^Cu-NODAGA-PSMA-I&T complexes.

RCP = radiochemical purity; t_R_ = retention time.

RCP and t_R_ refer to radio–high-performance liquid chromatography analysis. Lipophilicity (log*D*) was determined in 1:1 mixture of octanol–to–phosphate-buffered saline at pH 7.4. IC_50_ values were determined in competition assays on LNCaP cells using ((*S*)-1-carboxy-5-(4-(-^125^I-iodo-benzamido)pentyl)carbamoyl)-l-glutamic acid at concentration of 0.2 nM. Internalized and surface-bound fractions refer to percentage of applied activity after 1 h of incubation of LNCaP cells with radiotracer at 37°C. All results are expressed as mean ± SD.

[^61^Cu]Cu-DOTAGA-PSMA-I&T and [^61^Cu]Cu-NODAGA-PSMA-I&T were synthesized with radiolabeling yield of more than 98% and radiochemical purity of more than 97% (radio–high-performance liquid chromatography analysis) at an apparent molar activity of 24 MBq/nmol. Thus, no purification was required after labeling. Although [^61^Cu]Cu-DOTAGA-PSMA-I&T needed elevated temperature (95°C) and 15 min of reaction time, [^61^Cu]Cu-NODAGA-PSMA-I&T was synthesized at room temperature within 5 min. After 4 h at room temperature, the radiochemical purity of [^61^Cu]Cu-DOTAGA-PSMA-I&T dropped to approximately 90%, whereas it remained stable (∼97%) for [^61^Cu]Cu-NODAGA-PSMA-I&T. All analytic data and quality control results are provided in Supplemental Figures 1 and 2 and in Supplemental Tables 1 and 2.

[^61^Cu]Cu-DOTAGA-PSMA-I&T and [^61^Cu]Cu-NODAGA-PSMA-I&T were more lipophilic (log*D* = −2.69 ± 0.44 and −2.95 ± 0.08, respectively; *P* = 0.005) than the reference tracers [^68^Ga]Ga-PSMA-11 (log*D* = −3.89 ± 0.19, *P* < 0.0001 for both ^61^Cu-labeled tracers) and [^18^F]PSMA-1007 (log*D* = −3.02 ± 0.11, *P* = 0.0008 for [^61^Cu]Cu-DOTAGA-PSMA-I&T and *P* = 0.0446 for [^61^Cu]Cu-NODAGA-PSMA-I&T) and showed similar lipophilicity to their ^68^Ga-counterparts (Supplemental Table 3).

**FIGURE 1.**
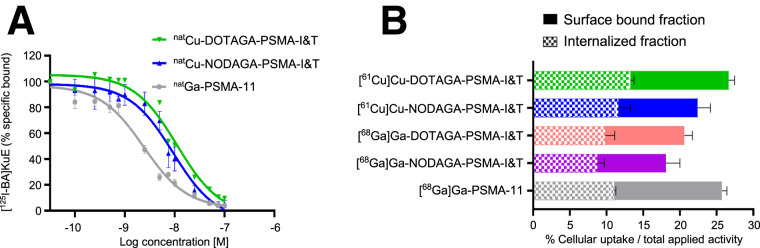
In vitro assessment in LNCaP cells after 1 h at 37°C. (A) Competition binding activity curves of ^nat^Cu-DOTAGA-PSMA-I&T, ^nat^Cu-NODAGA-PSMA-I&T, and ^nat^Ga-PSMA-11 on LNCaP cells after 1 h of incubation on ice, using ((*S*)-1-carboxy-5-(4-(-^125^I-iodo-benzamido)pentyl)carbamoyl)-l-glutamic acid ([^125^I-BA]KuE) at concentration of 0.2 nM as reference radioligand. (B) Cellular uptake (surface-bound + internalized). Cell surface–bound and internalized fractions are indicated. Results represent mean ± SD of specific (total − nonspecific) uptake from minimum of 2 separate experiments, each in triplicate.

The affinity of ^nat^Cu-DOTAGA-PSMA-I&T and ^nat^Cu-NODAGA-PSMA-I&T was in the low nanomolar range (IC_50_, 11.2 ± 2.3 and 9.3 ± 1.8 nM, respectively) and lower than the reference ^nat^Ga-PSMA-11 (IC_50_, 2.4 ± 0.4 nM) (Fig. 1A).

Cellular uptake (Fig. 1B) of [^61^Cu]Cu-DOTAGA-PSMA-I&T and [^61^Cu]Cu-NODAGA-PSMA-I&T after 1 h at 37°C (26.6% ± 1.3% and 22.4% ± 3.3%, respectively) was higher than that of their ^68^Ga counterparts, [^68^Ga]Ga-DOTAGA-PSMA-I&T (20.6% ± 2.3%, *P* = 0.0002) and [^68^Ga]Ga-NODAGA-PSMA-I&T (18.1% ± 2.6%, *P* = 0.0047), and similar to that of the reference [^68^Ga]Ga-PSMA-11 (25.8% ± 0.5%, *P* > 0.05 for both ^61^Cu-labeled tracers). All radiotracers were distributed almost equally between the surface (membrane)-bound and the internalized fraction. With time, the internalized fraction rose, whereas the surface-bound fraction remained relatively constant (Supplemental Table 4).

### Preclinical PET/CT Imaging

**FIGURE 2.**
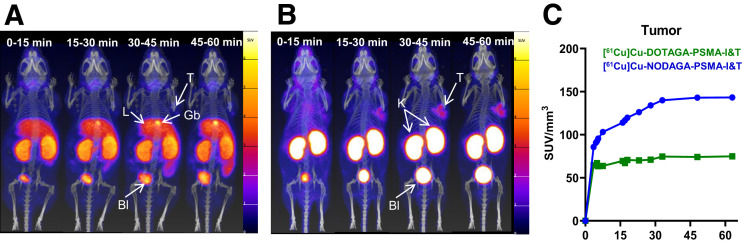
(A and B) Maximum intensity projections of dynamic PET/CT scans of LNCaP xenografts after injection of 400 pmol/7–8 MBq [^61^Cu]Cu-DOTAGA-PSMA-I&T (A) and [^61^Cu]Cu-NODAGA-PSMA-I&T (B) 0–1 h after injection, in 15-min frames. (C) Time–activity curves of tumor derived from dynamic PET/CT scans. Bl = bladder; Gb = gallbladder; K= kidneys; L = liver; T = tumor.

**FIGURE 3.**
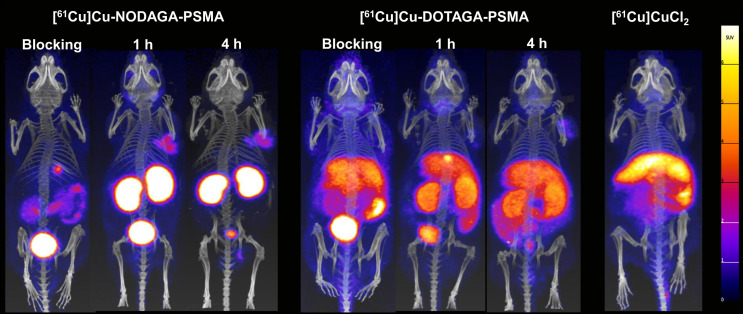
Maximum intensity projections of PET/CT scans at 1 and 4 h after injection of 100 µL/400 pmol/7–8 MBq [^61^Cu]Cu-NODAGA-PSMA-I&T or [^61^Cu]Cu-DOTAGA-PSMA-I&T in LNCaP xenografts. Blocking represents PET/CT scans 1 h after injection of mice preinjected with 2-(phosphonomethyl)pentanedioic acid (100 µL/1.3 µmol). PET/CT image of [^61^Cu]CuCl_2_ (7 MBq) is provided for comparison.

Figures 2A and 2B show the dynamic PET/CT scans of [^61^Cu]Cu-DOTAGA-PSMA-I&T and [^61^Cu]Cu-NODAGA-PSMA-I&T, respectively, from 0 to 1 h after injection, and Figure 3 compares PET/CT imaging of an early versus a late time point (1 vs. 4 h after injection). [^61^Cu]Cu-DOTAGA-PSMA-I&T accumulated mainly in the liver, kidneys, intestine, and gallbladder. Undesirable accumulation in the abdomen, especially the liver and intestines, was predominant at 4 h after injection. In contrast, [^61^Cu]Cu-NODAGA-PSMA-I&T showed a favorable biodistribution profile with renal accumulation and persistent tumor uptake between 1 and 4 h after injection. Time–activity curves (Fig. 2C) showed uptake in the tumor within minutes after injection, an uptake peak of approximately 30 min, and stability remaining for up to 60 min.

Specificity studies (Fig. 3) illustrated that uptake in PSMA-positive LNCaP tumors, kidneys, and salivary glands was significantly reduced after injection of 2-(phosphonomethyl)pentanedioic acid. However, liver and abdominal uptake of [^61^Cu]Cu-DOTAGA-PSMA-I&T was not affected. PET/CT imaging of [^61^Cu]CuCl_2_ suggested that this resulted from the release of ^61^Cu from the DOTAGA complex. Dynamic PET/CT images of [^61^Cu]CuCl_2_ (Supplemental Fig. 3) illustrated that uncomplexed ^61^Cu accumulated quickly in the liver and with time in the liver and intestine.

Between the 2 ^61^Cu-labeled PSMA tracers, [^61^Cu]Cu-NODAGA-PSMA-I&T presented the expected biodistribution profile of other PSMA-targeted radiotracers, such as [^68^Ga]Ga-PSMA-11, [^68^Ga]Ga-DOTAGA-PSMA-I&T, [^68^Ga]Ga-NODAGA-PSMA-I&T, and [^18^F]PSMA-1007 (Supplemental Fig. 4). This was not the case for [^61^Cu]Cu-DOTAGA-PSMA-I&T because of its high liver and abdominal uptake.

### Biodistribution Studies, Dosimetry, and In Vivo Metabolic Stability

The quantitative biodistribution data of [^61^Cu]Cu-DOTAGA-PSMA-I&T and [^61^Cu]Cu-NODAGA-PSMA-I&T are shown in Table 2. [^61^Cu]Cu-DOTAGA-PSMA-I&T had approximately 10-fold higher blood values and background activity with predominant and persistent accumulation in the liver and the abdomen, shown to be unspecific. [^61^Cu]Cu-NODAGA-PSMA-I&T was characterized by fast blood clearance, high kidney uptake, and minimal accumulation in PSMA-negative tissues compared with [^61^Cu]Cu-DOTAGA-PSMA-I&T. Tumor uptake was significantly higher for [^61^Cu]Cu-NODAGA-PSMA-I&T than for [^61^Cu]Cu-DOTAGA-PSMA-I&T at 1 h after injection (14.0 ± 5.0 vs. 6.06 ± 0.25 percentage injected activity per gram of tissue [%IA/g], *P* = 0.0059) and at 4 h after injection (10.7 ± 3.3 vs. 4.88 ± 0.63 %IA/g, *P* = 0.0014). [^61^Cu]Cu-NODAGA-PSMA-I&T had higher tumor-to-nontumor ratios than [^61^Cu]Cu-DOTAGA-PSMA-I&T (Table 3), with the exception of the tumor-to-kidney ratio at 4 h after injection.

**TABLE 2.** Biodistribution of [^61^Cu]Cu-DOTAGA-PSMA-I&T and [^61^Cu]Cu-NODAGA-PSMA-I&T vs. [^68^Ga]Ga-PSMA-11 and [^18^F]PSMA-1007 in LNCaP Xenografts

	[^61^Cu]Cu-DOTAGA-PSMA-I&T			[^61^Cu]Cu-NODAGA-PSMA-I&T			[^68^Ga]Ga-PSMA-11, 1 h	[^18^F]PSMA-1007	
Organ	1 h	1 h blocking	4 h	1 h	1 h blocking	4 h		1 h	4 h
Blood	2.06 ± 0.24	1.36 ± 0.15	1.12 ± 0.24	0.28 ± 0.06	0.19 ± 0.01	0.10 ± 0.03	0.25 ± 0.07	0.41 ± 0.11	0.17 ± 0.04
Heart	3.73 ± 0.32	2.34 ± 0.11	2.08 ± 0.33	0.45 ± 0.15	0.33 ± 0.03	0.22 ± 0.05	0.32 ± 0.10	1.25 ± 0.29	0.53 ± 0.23
Lung	6.35 ± 0.35	5.62 ± 1.61	4.67 ± 0.76	1.69 ± 0.45	0.67 ± 0.06	0.74 ± 0.19	1.43 ± 0.43	2.08 ± 0.24	1.61 ± 0.58
Liver	19.3 ± 3.3	14.3 ± 1.7	13.9 ± 2.2	1.02 ± 0.28	1.29 ± 0.21	0.72 ± 0.11	0.54 ± 0.27	0.93 ± 0.26	0.32 ± 0.16
Pancreas	2.82 ± 0.67	1.89 ± 0.34	1.71 ± 0.23	0.97 ± 0.36	0.28 ± 0.17	0.45 ± 0.09	0.70 ± 0.12	1.32 ± 0.58	0.80 ± 0.32
Spleen	4.64 ± 1.35	2.46 ± 0.47	2.95 ± 0.73	6.04 ± 1.87	0.33 ± 0.08	1.28 ± 0.39	6.38 ± 1.37	11.0 ± 1.1	8.33 ± 2.11
Stomach	7.77 ± 0.62	7.52 ± 1.58	7.10 ± 0.97	1.13 ± 0.20	0.62 ± 0.04	0.66 ± 0.15	0.69 ± 0.11	0.75 ± 0.15	0.47 ± 0.16
Intestine	8.95 ± 0.62	8.78 ± 2.18	7.74 ± 1.76	2.11 ± 0.78	0.87 ± 0.10	1.06 ± 0.50	1.52 ± 0.60	1.04 ± 0.33	0.43 ± 0.22
Adrenals	9.35 ± 1.50	3.25 ± 0.92	6.46 ± 2.38	17.3 ± 3.26	0.95 ± 0.16	8.32 ± 2.87	19.2 ± 5.3	7.18 ± 2.20	8.03 ± 2.66
Kidneys	57.1 ± 6.3	8.76 ± 1.57	22.1 ± 2.2	118 ± 13	4.59 ± 0.35	90.9 ± 10.1	159 ± 31	100 ± 17	132 ± 9
Muscles	1.00 ± 0.05	0.63 ± 0.10	0.48 ± 0.08	1.12 ± 0.32	0.32 ± 0.06	0.50 ± 0.22	0.90 ± 0.36	0.53 ± 0.09	0.27 ± 0.11
Femur	2.31 ± 0.36	1.50 ± 0.21	1.69 ± 0.25	2.73 ± 0.91	0.39 ± 0.24	1.34 ± 0.48	3.69 ± 1.86	0.94 ± 0.13	0.62 ± 0.11
Salivary glands	5.60 ± 1.39	3.25 ± 0.94	2.31 ± 0.19	2.01 ± 0.33	0.51 ± 0.20	0.52 ± 0.06	1.56 ± 0.31	2.54 ± 0.72	1.62 ± 0.43
Tumor	6.06 ± 0.25	3.31 ± 0.26	4.88 ± 0.63	14.0 ± 5.0	0.68 ± 0.55	10.7 ± 3.3	10.2 ± 1.5	9.70 ± 2.57	6.28 ± 2.19

Results are expressed as mean of %IA/g ± SD of *n* = 4–8 mice per group.

**TABLE 3.** Tumor-to-Nontumor Ratios of [^61^Cu]Cu-DOTAGA-PSMA-I&T and [^61^Cu]Cu-NODAGA-PSMA-I&T vs. [^68^Ga]Ga-PSMA-11 and [^18^F]PSMA-1007 in LNCaP Xenografts Based on Biodistribution Data

	[^61^Cu]Cu-DOTAGA-PSMA-I&T		[^61^Cu]Cu-NODAGA-PSMA-I&T		[^68^Ga]Ga-PSMA-11, 1 h	[^18^F]PSMA-1007	
Organ	1 h	4 h	1 h	4 h		1 h	4 h
Blood	2.97 ± 0.23	4.56 ± 1.49	55.8 ± 20.0	109 ± 43	45.3 ± 19.7	25.8 ± 11.5	42.7 ± 16.4
Liver	0.32 ± 0.05	0.35 ± 0.05	14.9 ± 4.9	15.4 ± 5.7	23.5 ± 12.5	11.3 ± 4.5	18.4 ± 8.5
Spleen	1.37 ± 0.29	1.73 ± 0.46	2.86 ± 1.37	9.16 ± 3.84	1.69 ± 0.58	0.89 ± 0.20	0.71 ± 0.12
Intestine	0.68 ± 0.07	0.66 ± 0.23	7.87 ± 2.93	12.5 ± 6.7	8.66 ± 6.90	10.3 ± 4.8	12.4 ± 4.4
Adrenals	0.66 ± 0.13	0.81 ± 0.22	0.92 ± 0.20	1.44 ± 0.65	0.58 ± 0.26	1.45 ± 0.55	0.78 ± 0.11
Kidneys	0.11 ± 0.01	0.22 ± 0.04	0.13 ± 0.03	0.12 ± 0.03	0.07 ± 0.02	0.10 ± 0.03	0.04 ± 0.01
Muscles	6.08 ± 0.22	10.5 ± 2.8	13.6 ± 3.75	25.4 ± 12.0	13.6 ± 8.3	19.2 ± 6.5	20.1 ± 2.5
Salivary glands	1.14 ± 0.34	2.13 ± 0.43	7.83 ± 3.16	19.5 ± 7.1	6.72 ± 1.51	4.12 ± 1.60	3.63 ± 0.40

Results are expressed as mean ± SD of *n* = 4–8 mice per group.

Biodistribution studies at 1 h after injection confirmed the similar in vivo profile of [^61^Cu]Cu-NODAGA-PSMA-I&T with [^68^Ga]Ga-NODAGA-PSMA-I&T and [^68^Ga]Ga-DOTAGA-PSMA-I&T (Supplemental Table 5) and with the clinically used tracers [^68^Ga]Ga-PSMA-11 and [^18^F]PSMA-1007 (Table 2), with minor exceptions (e.g., higher spleen uptake for [^18^F]PSMA-1007). No significant difference was found in the tumor uptake of [^61^Cu]Cu-NODAGA-PSMA-I&T versus [^68^Ga]Ga-PSMA-11 (14.0 ± 5.0 vs. 10.2 ± 1.5 %IA/g, *P* = 0.0972) and [^18^F]PSMA-1007 (14.0 ± 5.0 vs. 9.70 ± 2.57 %IA/g, *P* = 0.080). At the later time point of investigation (4 h after injection), [^61^Cu]Cu-NODAGA-PSMA-I&T was compared only with [^18^F]PSMA-1007 because of the t_1/2_ of the radionuclides. [^61^Cu]Cu-NODAGA-PSMA-I&T had higher tumor uptake than [^18^F]PSMA-1007 (10.7 ± 3.3 vs. 6.28 ± 2.19 %IA/g, *P* = 0.0145) and better tumor-to-blood and tumor-to-nontumor ratios in most cases (Table 3).

[^61^Cu]Cu-NODAGA-PSMA-I&T was significantly more stable in vivo at 1 h after injection than [^61^Cu]Cu-DOTAGA-PSMA-I&T, which showed approximately 70% release of ^61^Cu in the liver (Supplemental Fig. 5). The pharmacokinetic data of [^61/64^Cu]Cu-NODAGA-PSMA-I&T 1–24 h after injection are provided in Supplemental Table 6 and were used for the dosimetry estimates. Table 4 shows the estimated radiation dose of [^61^Cu]Cu-NODAGA-PSMA-I&T for men, with an effective dose of 0.0142 mSv/MBq.

**TABLE 4.** Total Absorbed Doses in Different Organs of [^61^Cu]Cu-NODAGA-PSMA-I&T Calculated by OLINDA/EXM Version 1.0, with Assumption That Kinetics in Mouse Is Same as Kinetics in Human

Target organ	Total absorbed dose, men (mGy/MBq)
Adrenals	8.39E−02
Brain	6.18E−05
Breasts	—
Gallbladder wall	2.30E−02
LLI wall	4.64E−03
Small intestine	3.79E−02
Stomach wall	1.71E−02
ULI wall	1.43E−02
Heart wall	6.94E−03
Kidneys	1.74E+00
Liver	2.51E−02
Lungs	6.11E−03
Muscle	6.00E−03
Ovaries	6.37E−03
Pancreas	4.83E−02
Red marrow	1.11E−02
Osteogenic cells	5.31E−03
Skin	2.90E−03
Spleen	7.37E−02
Testes	5.39E−04
Thymus	1.67E−03
Thyroid	4.99E−04
Urinary bladder wall	2.06E−03
Uterus	—
Total body	1.38E−02
Effective dose (mSv/MBq)	1.42E−02

LLI = lower large intestine; ULI = upper large intestine.

Phantom was standard adult man.

### First-in-Human PET/CT Imaging

**FIGURE 4.**
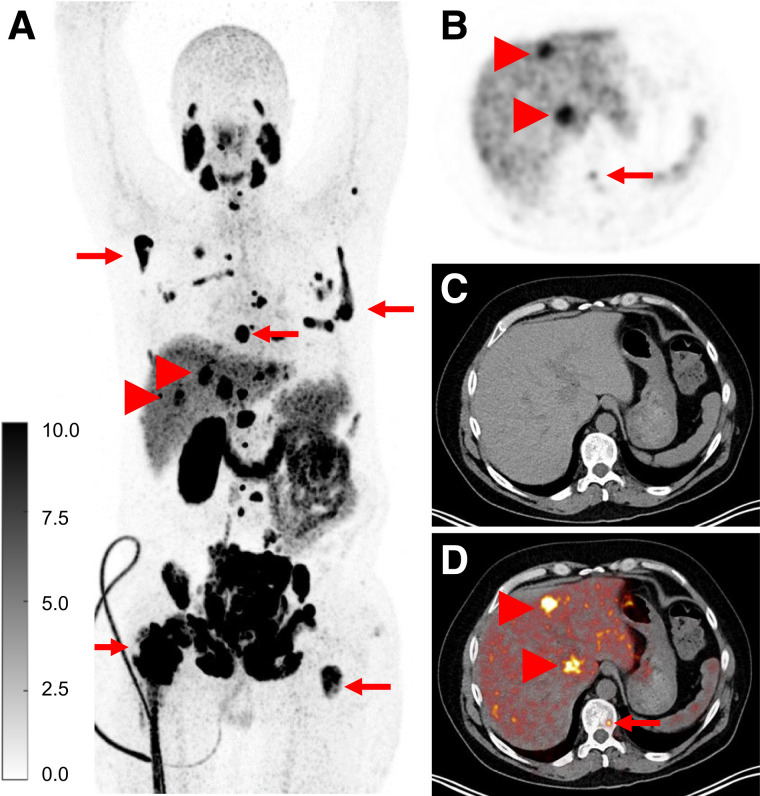
Administration of [^61^Cu]Cu-NODAGA-PSMA-I&T, in 48-y-old man with metastatic castration-resistant prostate cancer with disease progression, after abiraterone and docetaxel therapy before ^177^Lu-labeled PSMA radiopharmaceutical therapy. Imaging was performed 3 h after tracer administration. (A–D) Maximum intensity projection (A), and PET (B), CT (C), and fused PET/CT (D) images demonstrate multifocal osseous metastases (arrows) and hepatic metastases (arrowheads). Patient has also 1 kidney after left nephrectomy.

Administration of [^61^Cu]Cu-NODAGA-PSMA-I&T (specifications are provided in Supplemental Table 7) and imaging were performed (Fig. 4). Radiotracer accumulation was noted in multifocal osseous and hepatic metastases, and the physiologic distribution of PSMA-targeted tracers was as expected in the lacrimal glands, salivary glands, liver, spleen, kidneys, ureters, bladder, and proximal small bowel. The SUV_max_ and SUV_mean_ were as follows: salivary gland (right parotid gland), 20.5 and 12.6; liver, 6.5 and 3.4; and kidney (right), 57.3 and 37.9, respectively. For the tumor lesions, the SUV_max_ and SUV_mean_ were as follows: right lower pubic bone, 154.2 and 83.7; right scapula, 86.4 and 55.3; left liver lobe, 23.6 and 11.7; and sacral bone, 16.5 and 9.9, respectively.

## DISCUSSION

PSMA-targeted PET imaging has become a new standard of care for patients with prostate cancer (4*,*^17^*,*^18^). This study aimed to assess the feasibility of ^61^Cu-PSMA–targeted tracers in terms of performance, clinical relevance, ease of production, and accessibility. We aimed to provide insights into the advantages of this tracer compared with others in its class.

In vivo, [^61^Cu]Cu-NODAGA-PSMA-I&T showed clear superiority over [^61^Cu]Cu-DOTAGA-PSMA-I&T by means of higher tumor uptake (*P* = 0.0050 1 h after injection vs. *P* = 0.0066 4 h after injection), lower blood-pool activity, and especially liver and abdominal activity. The metabolic instability of [^61^Cu]Cu-DOTAGA-PSMA-I&T and the release of ^61^Cu from the DOTAGA chelator led to activity accumulation in the liver. Similar findings were shown previously in vivo with [^64^Cu]Cu-PSMA-617, in which DOTA was used as a chelator (19). Other ^64^Cu-labeled PSMA ligands reported in the literature are using more suitable chelators for Cu(II) than DOTA, such as cyclams (20) or sarcophagine (21), and different Glu-urea-Lys motifs. Among them, the dimer [^64^Cu]Cu-sarcophagine-bisPSMA is in a phase 3 trial (NCT06056830). To our knowledge, the conjugate NODAGA-PSMA-I&T under investigation has not been reported.

Overall, our in vivo studies with [^61^Cu]Cu-NODAGA-PSMA-I&T show biodistribution similar to that of [^68^Ga]Ga-PSMA-11 and [^18^F]PSMA-1007 at 1 h after injection. At the later time of 4 h after injection, [^61^Cu]Cu-NODAGA-PSMA-I&T had improved tumor-to-background ratios, demonstrating the advantage of using a longer-t_1/2_ radionuclide to optimize radiotracer biodistribution and tumor-to-background contrast. Dosimetry estimates of [^61^Cu]Cu-NODAGA-PSMA-I&T suggested that dosimetry is within the expected levels of the ^68^Ga-labeled and ^18^F-labeled PSMA tracers (22*,*^23^). Higher tumor-to-background ratios obtained in scans at later time points with [^61^Cu]Cu-NODAGA-PSMA-I&T can potentially enhance the detection rate of lesions and provide clarification of findings that were unclear in scans at early time points. This observation is supported by several studies that have shown increased PSMA lesion detection rates when scans are performed beyond the initial 1-h window using various tracers, such as [^68^Ga]Ga-PSMA (22*,*^24^*,*^25^), ^18^F-labeled PSMA tracers (26), or more recently ^99m^Tc-labeled PSMA tracers (27).

This work takes the newly developed ^61^Cu-PSMA-targeted tracer into successful first-in-human imaging. Although a single subject does not guarantee future success, we are highly encouraged by the prominent tracer uptake in both osseous and hepatic metastases, which are clearly visualized.

Most PSMA-targeted PET tracers use ^68^Ga and ^18^F for radiolabeling. Thus far, the implementation of these tracers has been constrained by the relatively short t_1/2_ of ^68^Ga and ^18^F, which restricts the efficient distribution of tracers beyond a limited geographic range and the availability of delayed imaging. Centralized production facilities of ^18^F-labeled tracers are generally confined to distribution areas of a few hundred miles, requiring substantial networks of production facilities. Even with multiple production sites, wide areas of the population may not be able to receive and use these radionuclides.

Copper radioisotopes are attractive for use in both molecular imaging and therapy, because positron-emitting ^61^Cu (t_1/2_, 3.33 h) and ^64^Cu (t_1/2_, 12.7 h) agents may be paired with β^−^-emitting ^67^Cu agents to create true theranostic pairs. ^64^Cu has been used previously in PET imaging tracers such as [^64^Cu]Cu-DOTATATE, given its commercial availability (28). ^64^Cu has a longer t_1/2_ (12.7 h) than ^68^Ga and ^18^F, allowing greater geographic distribution of products and delayed imaging. However, ^64^Cu is limited by its low positron yield (18%), which may impair image quality, and 39% of its decays are β^−^-emissions, increasing radiation exposure. Compared with ^64^Cu, ^61^Cu combines the advantage of long t_1/2_ with far greater positron yield (61%) yet lacks high-energy β^−^-emissions (29). The physical properties of ^61^Cu versus ^64^Cu are compared in Supplemental Table 8.

Despite the favorable physical properties of ^61^Cu, the literature has reported only a few instances of ligands labeled with this radionuclide (30–32). This scarcity can be attributed primarily to the limited availability and distribution of ^61^Cu. However, recent advances in the automated cyclotron production of [^61^Cu]CuCl_2_ using liquid zinc (13) and solid nickel targets (14) have paved the way for greater accessibility to ^61^Cu and subsequently expanded its potential for clinical applications (10).

With inexpensive ^nat^Ni as the starting material, highly pure ^61^Cu could be produced with radionuclidic purity exceeding 99.99% at 12 h after synthesis (details on the production will be published elsewhere). The preparation of [^61^Cu]Cu-NODAGA-PSMA-I&T was performed in widely used buffers at room temperature within 5 min. The radiolabeling process at the apparent molar activity of 24 MBq/nmol demonstrated high yield (>98%) and stability (≥97% up to 4 h). Moreover, [^61^Cu]Cu-NODAGA-PSMA-I&T synthesized in a good manufacturing practice grade for human use was found to be stable for up to 9 h at room temperature, at an activity concentration of 20 MBq/nmol (Supplemental Table 9). These results surpass the typical yields achieved with ^18^F-labeled PSMA tracers in the same class (33). High labeling yields and suitable molar activities for clinical use eliminate the need for any purification step after labeling. Furthermore, the production of ^61^Cu can be scaled up to meet the growing demand. ^61^Cu production necessitates 1–3 h of cyclotron beam time, and its yield varies from 3 to 100 GBq, depending on the enrichment of the starting nickel material (^60^Ni and ^61^Ni) and beam parameters. These are advantageous features compared with ^64^Cu production, which demands 4–12 h of beam time for a 3- to 10-GBq yield and necessitates highly enriched (>98%) ^64^Ni to achieve the necessary radionuclidic purity and specific activity. All of these factors play crucial roles in determining the ease of production, scalability, and overall viability of the tracer for practical implementation in clinical settings.

## CONCLUSION

This study demonstrates the successful development, in vitro and in vivo characterization, and first-in-human imaging of ^61^Cu-labeled tracers for PSMA targeting. [^61^Cu]Cu-NODAGA-PSMA-I&T had better biodistribution, pharmacokinetics, and imaging properties than [^61^Cu]Cu-DOTAGA-PSMA-I&T. It also compared favorably with [^68^Ga]Ga-PSMA-11 and [^18^F]PSMA-1007 and demonstrated advantages at delayed imaging times. The study highlights the straightforward production of a high-quality ^61^Cu-labeled PSMA-targeted tracer suitable for future implementation. Several factors, such as radiochemical yield, radiochemical purity, and stability, that significantly affect a PET tracer’s production, distribution, and clinical viability were also assessed. Imaging with [^61^Cu]Cu-NODAGA-PSMA-I&T successfully visualized multifocal metastatic prostate cancer. Overall, the findings of this study serve as a foundation for future clinical development of ^61^Cu-labeled tracers and suggest opportunities for development of other ^61^Cu-labeled tracers for a range of clinically valuable targets.

## DISCLOSURE

The study was financially support by the Swiss Innovation Agency (Innosuisse), project 37014.1 IP-LS, and by Nuclidium AG (matching funding). Melpomeni Fani reports research funding from Ipsen, ITM, and Nuclidium; acts as a scientific advisor of Nuclidium; and is coinventor on patent applications filed by Nuclidium and the University of Basel related to ^61^Cu-labeled tracers. Francesco De Rose and Leila Jaafar-Thiel are employees of Nuclidium and coinventors in a series of patents related to ^61^Cu-labeled tracers. Gary Ulaner discloses that he receives speaker fees and research support from Lantheus, GE Healthcare, and RayzeBio and serves on the scientific advisory boards of Lantheus, GE Healthcare, RayzeBio, and Nuclidium. Matthias Eiber reports fees from Blue Earth Diagnostics Ltd. (consultant and research funding), Novartis/AAA (consultant and speaker), Telix (consultant), Bayer (consultant and research funding), RayzeBio (consultant), Point Biopharma (consultant), Eckert-Ziegler (speaker), Janssen Pharmaceuticals (consultant and speakers bureau), Parexel (image review), and Bioclinica (image review) outside the submitted work. He and other inventors are entitled to royalties on sales of Posluma. Wolfgang Weber reports fees from Nuclidium, TRIMT, BMS, Ipsen, Imaginab, and Piramal (grants); RayzeBio, Bayer, Blue Earth Diagnostics, Pentixapharm, and Vida Ventures (consultant); GSK and AAA (speakers’ bureau); and ITM, Endocyte, and Reflexion (advisory board). No other potential conflict of interest relevant to this article was reported.

## Notes

KEY POINTS **QUESTION:** Do ^61^Cu-PSMA–targeted tracers have benefits compared with other PSMA-targeted tracers? **PERTINENT FINDINGS:** This preclinical study introduced ^61^Cu-labeled PSMA tracers and identified [^61^Cu]Cu-NODAGA-PSMA-I&T as a suitable radiotracer for PSMA PET imaging. [^61^Cu]Cu-NODAGA-PSMA-I&T compared favorably with the clinically used [^68^Ga]Ga-PSMA-11 and [^18^F]PSMA-1007 and demonstrated advantages at delayed 4-h imaging. First-in-human imaging provided the proof of concept for the successful development and clinical translation of ^61^Cu-PSMA–targeted PET. **IMPLICATIONS FOR PATIENT CARE:** [^61^Cu]Cu-NODAGA-PSMA-I&T has benefits in terms of performance, ease of production, and accessibility and the potential to face the high demand for PSMA PET scans.
